# Quantifying energy expenditure in childhood: utility in managing pediatric metabolic disorders

**DOI:** 10.1093/ajcn/nqz177

**Published:** 2019-08-13

**Authors:** Laura P E Watson, Katherine S Carr, Michelle C Venables, Carlo L Acerini, Greta Lyons, Carla Moran, Peter R Murgatroyd, Krishna Chatterjee

**Affiliations:** 1 National Institute for Health Research (NIHR) Cambridge Clinical Research Facility, Addenbrooke's Hospital, Cambridge, United Kingdom; 2 Nutrition Surveys and Studies, Medical Research Council (MRC) Elsie Widdowson Laboratory, Cambridge, United Kingdom; 3 NIHR Biomedical Research Centre Nutritional Biomarker Laboratory, University of Cambridge, Cambridge, United Kingdom; 4 Department of Pediatrics, University of Cambridge, Addenbrooke's Hospital, Cambridge, United Kingdom; 5 University of Cambridge Metabolic Research Laboratories, Wellcome Trust–MRC Institute of Metabolic Science, Addenbrooke's Hospital, Cambridge, United Kingdom

**Keywords:** healthy boys and girls, resistance to thyroid hormone, dual-energy X-ray absorptiometry, indirect calorimetry, resting energy expenditure prediction equations

## Abstract

**Background:**

Energy expenditure prediction equations are used to estimate energy intake based on general population measures. However, when using equations to compare with a disease cohort with known metabolic abnormalities, it is important to derive one's own equations based on measurement conditions matching the disease cohort.

**Objective:**

We aimed to use newly developed prediction equations based on a healthy pediatric population to describe and predict resting energy expenditure (REE) in a cohort of pediatric patients with thyroid disorders.

**Methods:**

Body composition was measured by DXA and REE was assessed by indirect calorimetry in 201 healthy participants. A prediction equation for REE was derived in 100 healthy participants using multiple linear regression and *z* scores were calculated. The equation was validated in 101 healthy participants. This method was applied to participants with resistance to thyroid hormone (RTH) disorders, due to mutations in either thyroid hormone receptor β or α (β: female *n* = 17, male *n* = 9; α: female *n* = 1, male *n* = 1), with deviation of REE in patients compared with the healthy population presented by the difference in *z* scores.

**Results:**

The prediction equation for REE = 0.061 * Lean soft tissue (kg) − 0.138 * Sex (0 male, 1 female) + 2.41 (*R*^2^ = 0.816). The mean ± SD of the residuals is −0.02 ± 0.44 kJ/min. Mean ± SD REE *z* scores for RTHβ patients are −0.02 ± 1.26. *z* Scores of −1.69 and −2.05 were recorded in male (*n* = 1) and female ( *n* = 1) RTHα patients.

**Conclusions:**

We have described methodology whereby differences in REE between patients with a metabolic disorder and healthy participants can be expressed as a *z* score. This approach also enables change in REE after a clinical intervention (e.g., thyroxine treatment of RTHα) to be monitored.

## Introduction

Predicting resting energy expenditure (REE) can be useful in, for example, assessing nutritional energy intake requirements in healthy subjects or patients, in circumstances where expertise or facilities to measure it accurately are not available. The most common published prediction equations used in a pediatric setting are by Schofield (1), Henry (2), Harris and Benedict (3), and Molnar et al. (4). These equations are based on characteristics such as age, sex, height, and weight and are derived from large diverse cohorts, often with pooled data (5, 6). Many studies have reported inaccuracies of current REE prediction equations based on traditional height and weight measurements, across a variety of ages, ethnicities, and disease populations (7–11). The most recent published equations based on body composition measurements relevant to the healthy childhood age range include those of Muller et al. (5), which derive coefficients from fat-free mass (FFM), fat mass (FM), and sex and explain 72% of the variance. However, these data are based on observations pooled from separate German databases collected over a period of 18 y and may not be appropriate for other geographical populations or their associated disease groups.

Metabolic disorders are often associated with altered body composition (12). Adult patients with disorders such as thyrotoxicosis, resistance to thyroid hormone (RTH), and lipodystrophy have previously been shown to exhibit differences in both body composition and energy expenditure (13–16) compared with healthy controls (12). These disorders are also prevalent in childhood but diagnosed more rarely.

RTHβ, a genetic disorder due to mutations in the thyroid hormone receptor (TR) β gene, is characterized by elevated circulating thyroid hormones (THs), nonsuppressed thyroid stimulating hormone concentrations, and variable resistance to hormone action in peripheral tissues. The clinical features of RTHβ are highly variable, with most individuals being asymptomatic in a compensated euthyroid state, whereas a subset exhibit features (e.g., tachycardia and weight loss) reflecting hyperthyroidism of TRα-expressing tissues. This unpredictable clinical phenotype makes management of RTHβ in childhood difficult (17). Mutations in TRα causing RTHα are rare, with 30 patients from 17 families having been reported worldwide, 13 of whom were children. The clinical features of RTHα (growth retardation and developmental delay) resemble those in untreated childhood hypothyroidism (18, 19), reflecting hormone resistance and a relative hypothyroid state in TRα-expressing tissues.

After diagnosis, TH (thyroxine) therapy is used to alleviate hypothyroid features in RTHα. Conversely, in RTHβ, treatment with triiodothyroacetic acid, a TH analog which acts centrally to reduce hormone concentrations but is relatively devoid of thyromimetic effects in peripheral tissues, is used to control peripheral features of thyrotoxicosis. In both disorders, biochemical markers are monitored frequently to assess treatment, but the role of serial physiological measurements (e.g., energy expenditure and body composition) in children with RTH has not yet been evaluated.

The aim of this research was to demonstrate the use of newly developed prediction equations for REE in healthy participants for the purpose of describing differences in REE between healthy participants and participants with a metabolic disorder (RTH patient cohort) by computation of a *z* score.

## Methods

### Participants

Two hundred and one healthy male and female participants aged between 6 and 16 y and free from disease and medications took part in the study between July 2014 and December 2016 (see **Supplemental Figure 1** for the participant flowchart). Twenty-five participants with RTHβ were recruited between August 2006 and April 2016 and 2 participants with RTHα were recruited and followed up between October 2010 and February 2017. After reading the relevant information leaflets and having the study fully explained, the participants then assented and parents consented to taking part in the study. The healthy participants were recruited locally through advertisements including radio and ethical approval was granted by the East of England–Cambridge South ethics committee (14/EE/092) and the Research and Development department at Addenbrooke's hospital (A093198) in Cambridge. Participants with RTH were recruited after referral to the Endocrine clinic, Addenbrooke's hospital, with metabolic measurements being conducted under either clinical auspices or a research protocol [ethical approval was granted by East of England–Cambridge Central ethics committee (98/154) and the Research and Development department at Addenbrooke's hospital (A06658) in Cambridge].

### Body composition

All participants arrived at the National Institute for Health Research Cambridge Clinical Research Facility (CRF) in the afternoon having eaten lunch. On arrival and after the consent process, the participants underwent clinical measurements including blood pressure, temperature, and electrocardiogram. Height was measured on a stadiometer and recorded to the nearest millimeter (SECA electronic stadiometer) and weight was measured on electronic scales to the nearest gram (Kern & Sohn GmbH).

The participants then completed a whole body DXA assessment for bone mass (BM) and body composition [FM and lean soft tissue (LST)] (GE iDXA, analyzed in version 16, enhanced mode).

### REE

The participants stayed overnight at the CRF, Cambridge. They were fed an energy-balanced meal at their usual dinner time, based on the Schofield (1) predictions for energy expenditure. Usual bedtimes and waking times for each participant were adhered to.

REE was measured 30 min after waking by indirect calorimetry using a ventilated hood (GEM Nutrition). The participants were asked to remain still, awake, and not interact with others for 40 min during the measurements. Gas measurements of the room were made for the first 10 min, followed by the ventilated hood measurement of the participant for 20 min with a further 10 min of room gas analysis at the end of the measurement. The room measurements were to account for any apparent gas exchange which might arise in the absence of the participant from changes in room air composition after the initial room air sample. The gas exchange measurements were then converted into energy equivalents using calculations by Elia and Livesey (20). Before each measurement, the calorimeter was calibrated using 1% carbon dioxide and 20.9% oxygen. Annually the indirect calorimeters undergo 3 types of quality assurance tests: a flow rate tolerance test (reading within 2% of the measured flow rate), an infusion of N_2_ and carbon dioxide respiratory quotient of 0.85 test (ranging from 0.84 to 0.9), and a tolerance of drying tube test (effectiveness reading + 15 mL/min *V̇*O_2_). Repeated measurements of REE using our GEM Nutrition instruments demonstrate a CV of 3.8% and least significant change of 0.5 kJ/min (0.72 MJ/d).

### Statistical analysis

Data are reported as means ± SDs. Nonparametric tests were used throughout the analysis. Spearman correlations were determined between all variables (height, weight, age, BMI, FM, LST, BM) and REE. Stepwise multiple regression analysis was conducted to develop a prediction equation for REE. The Mann–Whitney *U* test was used to determine significant difference in distribution and variables between the regression cohort and validation cohort. The difference between measured and predicted REE is described as the residual. When an individual residual is divided by the SD of all the residuals, this represents a cohort *z* score. Although we have not assigned an absolute threshold for defining whether an individual data point is outside of the healthy range, if the *z* score for the patient is ±1, then it suggests there is a 68% probability that the observation belongs in the healthy cohort. If the *z* score is ±2, then the probability falls to 5%, making it 95% likely that the observation is associated with a disorder. IBM SPSS Statistics for Windows version 22.0 was used for descriptive statistics, correlations, and regression analysis. GraphPad Prism version 6 was used for Bland–Altman analyses.

## Results

The total cohort of 201 healthy participants was randomly divided into a regression data set (*n* = 100) and a validation data set (*n* = 101). Descriptive statistics for both data sets are presented in **Table 1**. There were no significant differences in age, height, weight, BMI, FM, LST, BM, and REE between the model and validation data sets.

**TABLE 1:** Descriptive characteristics for the regression model cohort and the validation cohort^1^

	Age, y							
	Regression model cohort (*n* = 100)				Validation cohort (*n* = 101)			
	6–8	9–11	12–14	15–16	6–8	9–11	12–14	15–16
	(*n* = 10 M, 14 F)	(*n* = 11 M, 20 F)	(*n* = 11 M, 13 F)	(*n* = 15 M, 6 F)	(*n* = 7 M, 13 F)	(*n* = 19 M, 18 F)	(*n* = 9 M, 16 F)	(*n* = 12 M, 7 F)
Age, y	7.6 ± 0.9	10.5 ± 0.8	13.4 ± 1.0	15.9 ± 0.7	7.4 ± 0.8	10.3 ± 0.9	13.3 ± 1.0	16.0 ± 0.7
Height, m	1.26 ± 0.06	1.43 ± 0.07	1.61 ± 0.09	1.73 ± 0.08	1.27 ± 0.10	1.45 ± 0.10	1.63 ± 0.08	1.75 ± 0.09
Weight, kg	27.3 ± 5.1	36.4 ± 7.2	48.7 ± 8.2	60.9 ± 8.8	26.0 ± 4.1	39.2 ± 10.2	50.9 ± 8.8	63.9 ± 10.2
BMI, kg/m^2^	17.2 ± 2.3	17.8 ± 2.6	18.8 ± 2.6	20.2 ± 2.0	16.2 ± 2.0	18.4 ± 3.1	19.2 ± 2.4	21.0 ± 3.9
FM, kg	7.8 ± 3.1	10.7 ± 4.3	12.2 ± 4.2	13.1 ± 4.7	7.1 ± 2.0	11.6 ± 5.4	13.6 ± 4.6	15.2 ± 7.3
LST, kg	18.6 ± 2.5	24.6 ± 3.6	34.8 ± 5.9	45.6 ± 7.8	18.3 ± 3.0	26.4 ± 5.6	35.7 ± 6.3	46.6 ± 8.4
BM, kg	0.97 ± 0.15	1.34 ± 0.23	1.91 ± 0.39	2.51 ± 0.44	0.97 ± 0.13	1.40 ± 0.30	2.00 ± 0.41	2.60 ± 0.30
REE, kJ/min	3.41 ± 0.35	3.92 ± 0.42	4.44 ± 0.50	5.07 ± 0.73	3.31 ± 0.51	4.09 ± 0.62	4.54 ± 0.63	4.93 ± 0.81

1 Values are means ± SDs. BM, bone mass; FM, fat mass; LST, lean soft tissue; REE, resting energy expenditure.

### Regression analysis

Regression analysis was performed with REE as the dependent variable. The predictor variables included those variables that were significantly correlated with REE in the bivariate analysis. With REE as the dependent variable, height (*r* = 0.883, *P* < 0.001), age (*r* = 0.788, *P* < 0.001), FM (*r* = 0.464, *P* < 0.001), LST (*r* = 0.896, *P* < 0.001), and BM (*r* = 0.864, *P* < 0.001) were correlated and entered into the regression analysis. After stepwise elimination of nonsignificant contributors to the regression, the results showed that there were 2 models that significantly predicted REE (**Table 2**). Model 1 included LST and accounted for 81% of the variation in REE and model 2 included LST and sex which accounted for 81.6% of the variation: REE = 0.061 * Lean soft tissue (LST) (kg) − 0.138 * Sex (0 male, 1 female) + 2.41 (*R*^2^ = 0.816).

**TABLE 2:** Stepwise regression coefficients based on *n* = 100 for the prediction of resting energy expenditure^1^

Model	Variable	Coefficient	SE	Adjusted *R*^2^	Sig *F* Change	95% CI
1	LST	0.063	0.003	0.810	0.000	0.056, 0.069
	Constant	2.288	0.097			2.095, 2.481
2	LST	0.061	0.003	0.816	0.047	0.055, 0.067
	Sex	–0.138	0.069			–0.274, –0.002
	Constant	2.410	0.113			2.185, 2.634

1 Sex, 0 = male, 1 = female. LST, lean soft tissue; *R*^2^, adjusted *R*^2^ representing the fit of the model.

Prediction model 2 was then applied to the validation cohort (*n* = 101), showing a significant correlation with measured REE (*r* = 0.850, *P* < 0.001).

**FIGURE 1:**
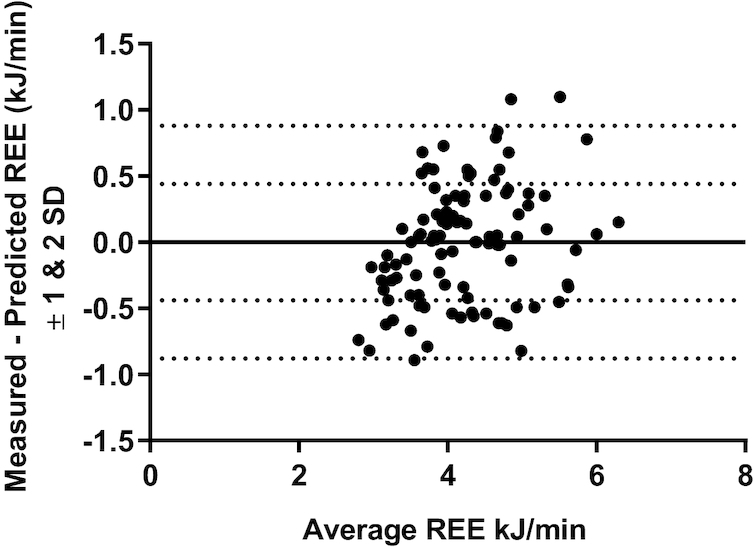
Bland–Altman agreement between measured and predicted REE in 101 healthy participants. Limits of agreement: −0.89 to 0.86; bias (mean ± SD): −0.02 ± 0.44. REE, resting energy expenditure.

The mean ± SD (95% CI) of the difference between measured and predicted REE was −0.02 ± 0.44 (−0.10, 0.07) kJ/min. The agreement between the measured REE and the predicted REE is presented by the Bland–Altman plot in **Figure 1**.

### Clinical application

Clinical and biochemical characteristics for RTHβ and RTHα participants are presented in **Table 3**.

**TABLE 3:** Descriptive characteristics of RTHβ and RTHα patients^1^

	RTHβ		RTHα	
	Male (*n* = 8)	Female (*n* = 17)	Male (*n* = 1)	Female (*n* = 1)
Height, m	1.39 ± 0.16	1.39 ± 0.19	1.52	0.99
Weight, kg	27.5 ± 8.7	39.8 ± 21.9	49.2	22.7
BMI, kg/m^2^	14.1 ± 2.2	19.5 ± 7.1	20.5	23.5
Age, y	10.7 ± 2.9	10.6 ± 3.8	15.5	5.8
FM, kg	3.7 ± 2.8	13.6 ± 12.5	8.4	4.6
LST, kg	22.7 ± 6.6	24.8 ± 9.4	39.1	17.5
BM, kg	1.1 ± 0.4	1.4 ± 0.8	1.7	0.7
TSH, mU/L (RR: 0.35–5.5)	3.3 ± 1.07	3.4 ± 1.07	2.07	1.04
FT4, pmol/L (RR: 9.01–22.7)	51.7 ± 30.2	46.1 ± 30.2	8.4	5.7
FT3, pmol/L (RR: 2.63–7.6)	19.1 ± 7.67	17.8 ± 7.67	9.1	6.9
REE, kJ/min	3.62 ± 0.55	3.86 ± 1.06	3.98	2.40

1 Normal RRs are based on age. BM, bone mass; FM, fat mass; FT3, triiodothyronine; FT4, thyroxine; LST, lean soft tissue; REE, resting energy expenditure; RR, reference range; RTH, resistance to thyroid hormone; TSH, thyroid stimulating hormone.

**FIGURE 2:**
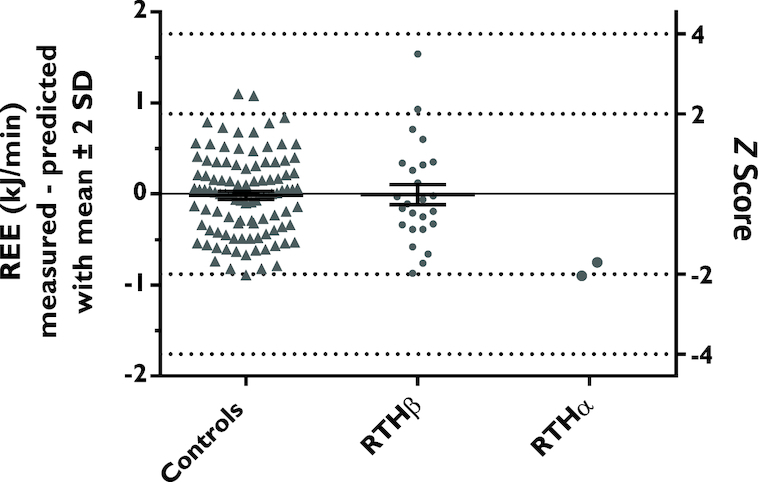
Residuals (mean ± SD) of measured and predicted REE and corresponding *z* scores for healthy (0.02 ± 0.44), RTHβ (−0.02 ± 1.26), and RTHα (male: −1.69; female: −2.05) groups. REE, resting energy expenditure; RTH, resistance to thyroid hormone.

The prediction equation (model 2) was then applied to participants with RTH disorders before treatment. The mean ± SD (95% CI) of the difference between measured and predicted REE (residuals) for RTHβ was −0.01 ± 0.55 (−0.24, 0.22) kJ/min. The RTHα participants exhibited lower energy expenditure for both the male and the female participants (difference: −0.75 kJ/min and −0.90 kJ/min, respectively) (**Figure 2**).

*z* Scores were then applied to the RTH cohorts based on the SD of the residuals derived from the healthy validation cohort (0.44). The mean ± SD *z* score for the RTHβ cohort was −0.02 ± 1.26. For the male and the female RTHα participant the *z* score was −1.69 and −2.05, respectively.

**FIGURE 3:**
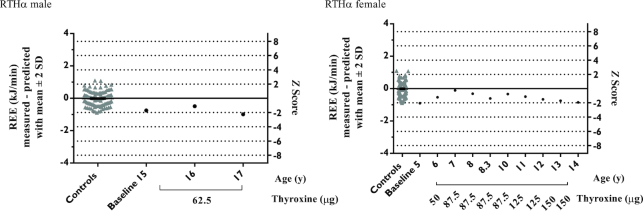
Residuals of measured and predicted REE and corresponding z scores at baseline and after treatment with thyroxine at the dosage (micrograms per day) indicated, in an individual male (left) and female (right) RTHα patient, compared with the healthy control cohort. REE, resting energy expenditure; RTH, resistance to thyroid hormone.

**Figure 3** shows the use of the prediction equations in monitoring serial changes in REE in individual male and female RTHα patients after thyroxine treatment, in comparison with healthy participants. For the male, at the age of 15 y, before treatment, the REE *z* score was −1.69 (mean difference −0.75 kJ/min), changing to −1.11 at age 16 y and −2.26 at 17 y (thyroxine dosage, 62.5 μg/d), after thyroxine therapy. For the female RTHα patient, the baseline REE *z* score at age 5 y, before thyroxine treatment, was −2.05. During thyroxine therapy (age 6–14 y), the REE *z* score changed, with values at ages 7 and 8 y being closest to zero (*z* score: −0.25 and −0.73; thyroxine dosage 87.5 μg/d) and from the age of 11 to 14 y reducing from −1.13 to −1.92, prompting further increases in thyroxine (125–150 μg/d).

## Discussion

The primary aim of this research was to develop prediction equations for REE in healthy participants aged 6–16 y, for the purpose of describing normal and disordered REE by the application of a *z* score in healthy individuals and also a cohort of RTH patients. The novelty of the concept lies in the derivation and application of a *z* score in an energy expenditure context. We described such an approach previously in an adult population (12), but its application to a pediatric population had not been evaluated hitherto, to our knowledge.

Typically, REE prediction equations have been derived based on specific populations of interest (e.g., children, obese, elderly, or disease groups) (7–11). Their purposes have been either for assessing energy intake requirements or for explaining changes in body composition such as weight loss or gain. This study proposes the use of prediction equations to describe and quantify deviation of REE in metabolic disorders from this physiological parameter in a healthy population.

Our prediction equations show that LST and sex are significant predictors explaining 81.6% of the variance in REE. Goran et al. (21) and Muller et al. (5) have also shown these variables to be determinants of REE, explaining 63% and 72% of the variation in REE in healthy nonobese children, respectively. When applying the Muller et al. equation to our validation data set, we observed a significant 10-fold difference in the mean ± SD residuals (0.27 ± 0.43 kJ/min compared with −0.02 ± 0.44 for Muller et al. and Watson et al., respectively). We would, however, expect there to be differences between the 2 prediction equations for 2 reasons; firstly, Muller et al. were only able to explain 72% of the variation in REE in 243 children, whereas our prediction equation explained 82% of the variation in 100 children. Secondly, there are methodological differences in the way REE data were collected.

One aim of generating childhood prediction equations was to apply them to metabolic disorders. The prediction equations were applied to disorders of TH action to describe the magnitude of difference in REE in patients compared with a healthy population. This approach has previously been used in adults (12), where thyrotoxic, lipodystrophic, and RTHβ patients all showed elevated energy expenditure *z* scores. Similarly, this study has identified differences in REE in 2 disorders, RTHβ and RTHα, in childhood. In RTHβ, patients showed a normal mean *z* score (−0.02) for REE, consistent with a compensated euthyroid state. In a previous study using published prediction equations (3, 4), Mitchell et al. (13) showed that REE was elevated ≤20% higher than predicted in both adults and children with RTHβ. The known phenotypic variability of RTHβ, with patients with differing degrees of resistance in peripheral tissues being recruited to each of these cohorts, may account for the observed difference in predicted REE in RTHβ between the 2 studies. In RTHα patients, low REE *z* scores (male *z* score: −1.69; female *z* score: −2.05) were documented, correlating with the known hypothyroid phenotype of the disorder and in agreement with observations reported previously (19, 22).

This study also illustrates the value of serial prediction equation–based measurements in individual patients with disordered metabolism. Treatment of a patient with conventional hypothyroidism with thyroxine would be expected to improve thyroid status and therefore increase REE. Serial measurement of REE *z* scores can provide an indirect assessment of the efficacy of thyroxine therapy. Advantages of depicting serial REE measurements as a *z* score, rather than as absolute values, are that REE has been adjusted for changes in body composition and is also compared with a matched healthy participant group.

Several limitations of this study should be considered. Firstly, for some age groups, the sample size was small. To apply the equations with confidence across the entire 6–16 y age range, larger sample sizes need to be studied. Secondly, this study did not assess a pubertal contribution to REE, whereas previously published prediction equations have shown an effect of pubertal stage (23). Lazzer et al. (24) proposed 2 REE prediction equations in obese children based on body mass or body composition. Their body composition equation was determined by the variables FFM, FM, sex, and pubertal stage using Tanner and Marshall scales (*R*^2^ = 0.70) (25, 26). The inclusion of pubertal stage in relation to REE by Lazzer et al. emphasizes the importance of using pubertal status rather than chronological age when investigating children with atypical body composition or REE.

In summary, body composition and REE have been described in a cohort of healthy participants and patients aged 6–16 y. Prediction equations for REE were developed using a healthy cohort, with *z* scores calculated in patients with rare disorders of TH action. Such novel use of REE *z* scores may facilitate assessment of energy expenditure in metabolic disorders and enable monitoring of responses to therapeutic or other intervention.

## Supplementary Material

nqz177_Supplemental_File

## Notes

Supported by the National Institute for Health Research (NIHR) Cambridge Clinical Research Facility, Cambridge, United Kingdom (to LPEW, KSC and PRM), Wellcome Trust grant 095564/Z/11/Z and the NIHR Cambridge Biomedical Centre (to KC and CM), and Medical Research Council Elsie Widdowson Laboratory program numbers Physiological Modelling of Metabolic Risk, MC_UP_A090_1005, and Nutrition, Surveys and Studies, MC_U105960384 (to MCV).

Data described in the article, code book, and analytic code will be made available upon request pending application and approval.

Supplemental Figure 1 is available from the “Supplementary data” link in the online posting of the article and from the same link in the online table of contents at https://academic.oup.com/ajcn/.

Abbreviations used: BM, bone mass; CRF, Clinical Research Facility; FFM, fat-free mass; FM, fat mass; LST, lean soft tissue; REE, resting energy expenditure; RTH, resistance to thyroid hormone; TH, thyroid hormone; TR, thyroid hormone receptor.
