# Supplementary material for: Quantifying energy expenditure in childhood: utility in managing pediatric metabolic disorders
Source: Am J Clin Nutr. 2019 Aug 13;110(5):1186–91. doi: 10.1093/ajcn/nqz177 (PMC6821543; doi:10.1093/ajcn/nqz177)
Supplement: nqz177_Supplemental_File [file nqz177_supplemental_file.docx]

Excluded (n=0)

289 participants requested study information sheet

201 healthy participants provided informed consent

Analysed datasets (n=201)

Excluded (n=23)

- Not meeting inclusion criteria (n=12)
- Declined to participate (n=9)
- Other reasons (n=2)

224 participants screened via questionnaire

65 participants dropped out

Supplemental Figure 1: Participant flow chart of the study.
